# Quality of Postoperative Pain Management in Orthopedic Patients and Its Impact on Sleep Quality and Patient Satisfaction: An Integrative Review

**DOI:** 10.7759/cureus.65872

**Published:** 2024-07-31

**Authors:** Omar M Alqaisi, Suhair Al-Ghabeesh

**Affiliations:** 1 Faculty of Nursing, Al-Zaytoonah University, Amman, JOR

**Keywords:** orthopedic surgery, postoperative care, patients satisfaction, sleep quality, pain management

## Abstract

Pain is one of the most common manifestations in the postoperative stage and it has a detrimental effect on both sleep and patient satisfaction. Consequently, this integrative review seeks to identify the outcomes of pain management specifically concerning sleep quality and patient satisfaction among the patients receiving orthopedic surgeries. In a stepwise manner, peer-reviewed articles manually searched in four databases including Scopus, Science Direct, PubMed, and CINAML (Cumulated Index to Nursing and Allied Health Literature) published between 2019 and 2023 were selected. The current review finally encompassed 22 studies. The review elaborates and reaffirms the notion that pain after surgery is still a critical issue that impacts the quality of patients’ sleep as well as their overall satisfaction. Chronic sleep disturbance is generally linked with pain while other factors such as light exposure and hospital environment were found to influence sleep quality. It is thus crucial to develop clear multifaceted pain management guidelines that include patient-tailored pharmacological and non-pharmacological interventions aimed at helping patients recover better, sleep better, and be satisfied with the procedures and results.

## Introduction and background

Pain is defined as an unpleasant experience that reduces the psychological and physical health of patients and has negative consequences such as discomfort, prolonged recovery, and the development of chronic pain [1,2]. About 300 million surgeries are performed for various reasons around the world annually [3]. However, pain after surgery is one of the most important complications that causes disability, long-term hospitalization, and financial burden [4-6]. Although protocols for postoperative pain management have evolved, pain remains an ongoing challenge to the medical community [7]. Studies indicate that pain management is affected by many factors such as gender, age, expectations before surgery, information given before surgery, type of anesthesia and medications given before surgery, and communication between staff and patients [8-10].

According to Kamphuis et al., sleep affects physical and mental health as it is considered one of the basic activities in a person's daily life [11]. Poor sleep quality is caused by several factors including psychological factors such as anxiety and stress, physical factors such as illness, severity of illness, and pain, and the hospital environment such as light and sound disturbances [12]. Sleep has an important role in inhibitory control of descending pain. Previous studies have also shown that high levels of pain after surgery, changes in behavior, and poor emotional health can lead to sleep disorders after surgery [13]. Sleep plays an important role in maintaining neuronal circuitry and maintaining overall health [14]. Various studies have found a bidirectional relationship between sleep disturbances, anxiety, and depression, suggesting that each contributes to development and is a consequence of the other [15]. The relationship between mood disorders, depression, and sleep quality remains unclear; however, sleep disturbances seem to be a risk factor for the development of depression. At the same time, depression and mood disorders are accompanied by sleep disturbances most of the time [16].

Patient satisfaction is a complex concept that is affected by many factors including economic, cultural, and social factors [17,18]. One of the pressing issues in the current field of medicine is patient satisfaction as it is an accepted measure of the quality of healthcare [19]. The overall level of patient satisfaction after anesthesia reported in different regions of the world ranges from 56.5% to 99.1% [20]. This creates large gaps between studies. Thus, this literature review focuses on postoperative pain management and its impact on sleep quality and patient satisfaction.

## Review

Method

This literature review was conducted following a systematic approach. The search was conducted on four databases Scopus, Science Direct, PubMed, and CINAHL (Cumulated Index to Nursing and Allied Health Literature). Once the initial pool of literature was identified, the next step involved screening. Inclusion criteria were defined as articles (i) published in English, (ii) published between 2019 and 2023, and (iii) full-text articles following quantitative, qualitative, or mixed-method designs conducted on adult populations only.

Search Outcome

**Figure 1:**
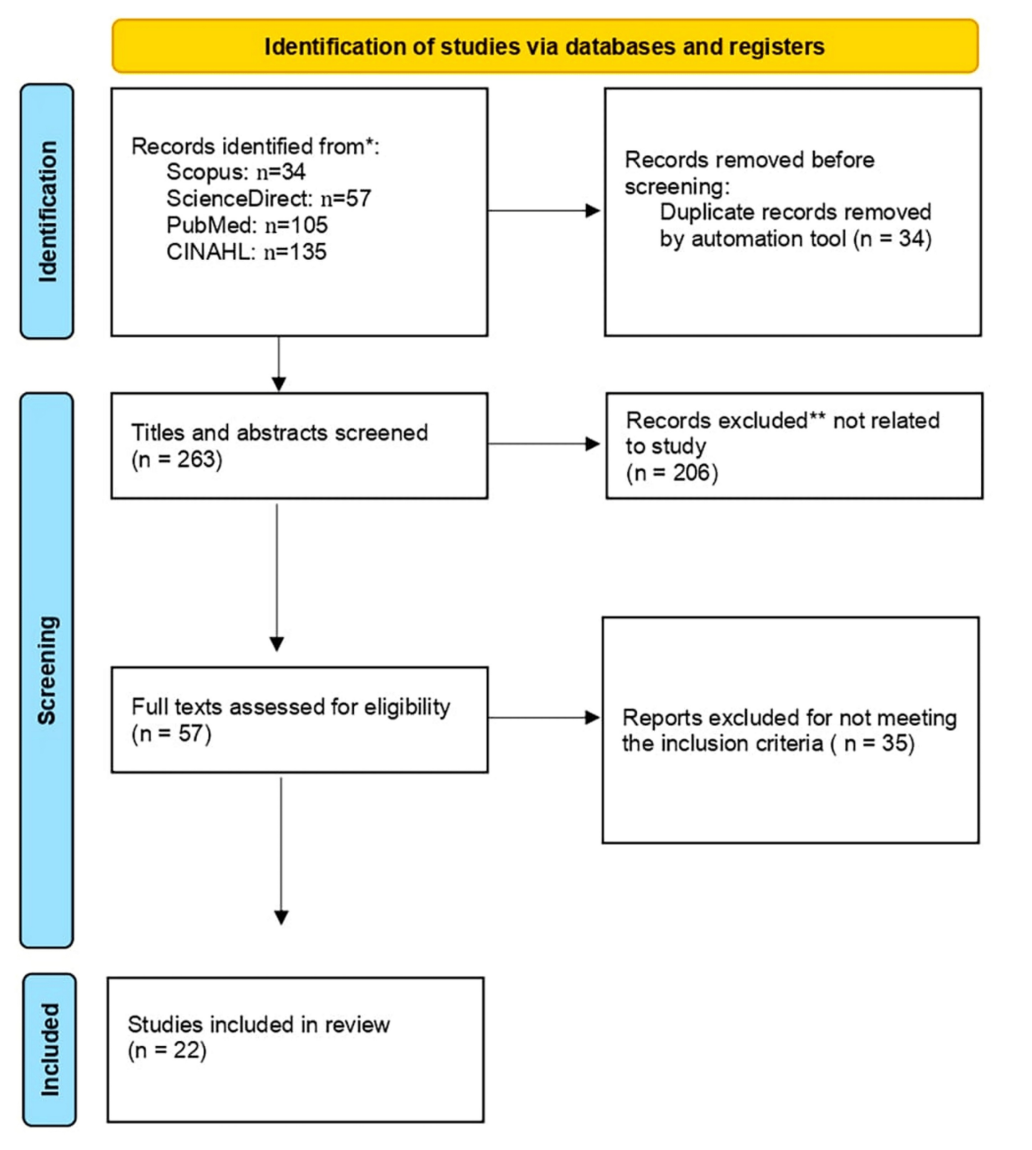
PRISMA flow diagram PRISMA: Preferred Reporting Items for Systematic Reviews and Meta-Analyses

The keywords used for the search were “postoperative pain”, "sleep quality", and "patient satisfaction" by using different combinations of Boolean operators. Only original research articles were included. Further filtering was applied to reach the search results presented in Figure 1.

Articles not related to the topic were excluded after reviewing titles and abstracts and only related articles are mentioned herein. All articles related to orthopedic surgeries that resulted from the search were reviewed. To our knowledge and according to the search results, none of the studies reported sleep quality and patient satisfaction together in relation to postoperative pain. This makes this review the first to address these factors together.

Data Abstraction

From the 22 that were included in the final literature review, the following data were abstracted and inserted into Table 1: author and year of publication, purpose of the study, country, sample size, design, and main findings. The process of selecting the final 22 studies is outlined in Figure and the studies are listed in Table 1.

**Table 1:** List of studies identified through search in different databases

Author	Purpose	Country	Sample Size	Design	Main Findings
Kulpatcharapong et al. (2020) [12]	To assess the prevalence and factors related to poor sleep quality in hospital patients.	Thailand	96	Observational analytic study	High prevalence of poor sleep quality among hospitalized patients, with improvements during the stay; light exposure and pain were key factors.
Song et al. (2020) [13]	To study how surgery timing affects anesthetic needs, postoperative sleep, and pain.	China	84	Comparative study	Evening surgeries required fewer anesthetics but resulted in more sleep disruption compared to daytime surgeries.
Fetene et al. (2022) [20]	To investigate perioperative surgical patient satisfaction and its predictors in three Ethiopian hospitals.	Ethiopia	411	Multicenter cross-sectional study	Overall low satisfaction with perioperative anesthesia services.
Mouch et al. (2020) [21]	To investigate hospital sleep quality, disruptions, and aids for improvement.	United States	195	Mixed-method sequential explanatory study	Most patients have poor sleep quality during recovery from elective surgery, with many disruptions being modifiable.
Shakya et al. (2019) [22]	To examine zolpidem's effect on postoperative sleep quality, pain relief, and life quality in hip surgery patients.	China	160	Randomized double-blind controlled trial	Zolpidem improved pain relief, early mobility, reduced anxiety and depression, and enhanced overall perioperative experience and satisfaction.
Fu et al. (2022) [23]	To explore tramadol's impact on postoperative mood, anxiety, and sleep.	China	200	Randomized controlled trial	Tramadol effectively improved mood and sleep post-surgery, supporting its use in postoperative pain management.
Xiao et al. (2022) [24]	To evaluate preoperative zolpidem's impact on analgesia use and postoperative pain.	China	88	Randomized prospective study	Preoperative zolpidem reduced intraoperative analgesic use and postoperative pain.
Lee et al. (2022) [25]	To compare intermittent epidural bolus injections versus continuous injections for nighttime pain and sleep quality.	Korea	76	Comparative study	Intermittent epidural bolus injections reduced nighttime pain and improved sleep quality more effectively than continuous injections.
Shen et al. (2021) [26]	To assess improved perioperative sleep's effect on pain, analgesic use, nausea and vomiting after knee or hip surgery.	Australia	1285	Systematic review and meta-analysis	Better perioperative sleep significantly reduced early postoperative pain and analgesic consumption without increasing nausea and vomiting.
Luo et al. (2019) [27]	To explore the correlation between preoperative sleep quality and postoperative outcomes in joint arthroplasty patients.	China	994	Prospective cohort study	Better preoperative sleep quality improved postoperative pain threshold, reduced pain scores and analgesic use, and shortened hospital stays.
Boye Larsen et al. (2021) [28]	To examine the relationship between preoperative sleep quality, pain, anxiety, depression and chronic postoperative pain after joint arthroplasty.	Denmark	185	Secondary analysis of a randomized controlled trial	Poor preoperative sleep correlated with higher pain, anxiety, and depression, leading to worse chronic postoperative pain outcomes.
Liu et al. (2023) [29]	To assess postoperative pain management efficacy in China and identify factors contributing to suboptimal pain control.	China	26193	Cross-sectional study	Almost half of the patients experienced moderate-to-severe pain post-surgery; high rate of opioid use was reported.
Berkowitz et al. (2019) [30]	To measure the link between patient-reported satisfaction, regret, and clinical outcomes.	United States	9953	Retrospective study	Significant link between clinical outcomes and satisfaction; postoperative pain had the most impact on satisfaction and regret.
Peng et al. (2019) [31]	To implement a comprehensive perioperative pain management program and assess its impact on recovery and satisfaction.	China	361	Controlled pilot study	Stratified pain management based on risk assessment improved pain control and postoperative recovery.
Garvey et al. (2021) [32]	To evaluate postoperative opioid use after arthroscopic rotator cuff repair.	United States	117	Prospective cohort study	Effective pain control achieved with a multimodal approach after arthroscopic rotator cuff repair.
Terro et al. (2022) [33]	To present the results of a novel approach to laparoscopic cholecystectomy for potential adoption.	Saudi Arabia	125	Retrospective study	High satisfaction with pain control and cosmetic outcomes following the new laparoscopic cholecystectomy method.
Yunus et al. (2020) [34]	To report on perceptions and satisfaction with postoperative pain management in emergency surgeries.	Nigeria	891	Prospective study	Low satisfaction with pain management in emergency surgeries.
Belay Bizuneh et al. (2020) [35]	To assess patient satisfaction with postoperative pain management and identify associated factors.	Ethiopia	43	Cross-sectional study	Low satisfaction with pain management; key factors included ASA status and immediate postoperative pain presence.
Xu et al. (2024) [36]	To compare outcomes of patients discharged with opioid versus opioid-free pain management after surgery.	New Zealand	4273	International multicenter cohort study	Opioid prescriptions at discharge did not improve satisfaction but increased healthcare visits due to side effects.
Stambough et al. (2021) [37]	To identify factors influencing the number of postoperative narcotic refills requested after joint arthroplasty.	United States	438	Retrospective cohort study	More narcotic refills did not correlate with improved satisfaction; preoperative opioid and benzodiazepine use predicted prolonged narcotic use.
Benavent et al. (2020) [38]	To examine patient satisfaction with pain management using an opioid prescription protocol after hand surgery.	United States	100	Prospective survey	Patients took fewer opioids than prescribed; higher pain scores were linked to lower satisfaction with opioid use.
Alema et al. (2023) [39]	To evaluate determinants of patient satisfaction with postoperative pain management in a specialized Ethiopian hospital.	Ethiopia	144	Cross-sectional study	Highlighted the need for in-depth studies on satisfaction and the inclusion of non-pharmaceutical pain management strategies.

Results

Characteristics of the Reviewed Studies

The reviewed studies encompass a diverse range of methodologies and focus areas related to postoperative pain, sleep quality, and patient satisfaction with pain management. A total of 22 studies were selected based on stringent inclusion criteria. These studies employed various research designs, including explanatory sequential mixed-method, analytic observational, prospective randomized controlled trials, systematic reviews, and meta-analyses. The sample sizes of the studies ranged from 43 to 26,193 participants, and they were conducted across multiple countries, including the United States, China, Australia, Ethiopia, and Denmark. The primary focus of these studies varied, but they all explored aspects of postoperative pain management, its impact on sleep quality, and patient satisfaction.

Postoperative Pain and Sleep Quality

The reviewed studies consistently indicate that postoperative pain significantly affects sleep quality in patients recovering from surgery. Mouch et al. found that most patients do not sleep well while recovering from elective surgery with most sleep disruptions being modifiable [21]. Similarly, Kulpatcharapong et al. highlighted a high prevalence of poor sleep quality in hospitalized patients which was partly improved during hospitalization [12]. Factors such as light exposure and pain were significant contributors to poor sleep quality. Studies by Shakya et al. [22], Fu et al. [23], and Xiao et al. [24] focused on pharmacological interventions to improve sleep quality and manage pain. Shakya et al. demonstrated that zolpidem, a sedative, can relieve pain, increase early range of motion and muscle strength, reduce perioperative anxiety and depression, and improve perioperative experience and satisfaction. Similarly, Fu et al. found that intravenous analgesia using tramadol effectively improved postoperative depression and sleep status in women undergoing abdominal endoscopic surgery [23]. Xiao et al. reported that improving preoperative sleep quality with zolpidem reduced intraoperative analgesic use and postoperative pain [24].

Lee et al. [25] and Shen et al. [26] investigated different pain management techniques and their impact on sleep quality. Lee et al. observed that programmed intermittent epidural bolus injections were more effective in reducing nighttime pain and improving sleep quality compared to continuous epidural injections [25]. Shen et al. conducted a systematic review and meta-analysis that confirmed improved perioperative sleep could significantly reduce pain levels at the early stage after total knee or hip arthroplasty (TKA or THA), decreasing analgesic drug consumption without increasing the incidence of postoperative nausea and vomiting [26].

Timing of surgery and its impact on sleep quality and pain was explored by Song et al., who reported that evening operations required lower dosages of anesthetic drugs but greater sleep disruption occurred when anesthesia and surgery were performed at night [13]. Luo et al. highlighted that better sleep quality before the operation improves pain threshold decreases pain score and analgesic consumption and reduces the length of stay (LOS) [27]. Studies by Boye et al. [28] and Liu et al. [29] examined the broader implications of sleep quality on pain and overall recovery. Boye et al. noted that patients with poor preoperative sleep have higher preoperative pain intensities and higher levels of pain, catastrophizing anxiety and depression [28]. Liu et al. reported that almost half of the patients suffered from moderate-to-severe pain after surgery in China with a high rate of systemic opioid use [29].

Patient Satisfaction with Postoperative Pain Management

Patient satisfaction with pain management is influenced by various factors including the effectiveness of pain control methods and the management of side effects. Berkowitz et al. found a strong association between patients' clinical outcomes and their satisfaction with postoperative pain being a critical determinant [30]. As reported by several researchers, personalized pain management approach has a role in increasing patient satisfaction. In a study by Peng et al., it was shown that a stratified approach to pain management through risk assessment enhanced the efficacy of analgesics thereby improving postoperative recovery and patient satisfaction [31]. Satisfactory pain control was achieved by Garvey et al. with a multimodal approach in patients following arthroscopic rotator cuff repair in 2021 [32]. The postoperative satisfaction of the patient with pain control and cosmetic appearance in a new approach to laparoscopic cholecystectomy was very much beyond expectation as unraveled by Terro et al. [33]. On the other hand, patient satisfaction seems very low in some regions based on studies conducted by Yunus et al. [33], Liu et al. [29], and Belay Bizuneh et al. [35], calling for improved standards in the current pain management protocols. Yunus et al. [34] noted dissatisfaction in pain management specifically among subjects who had gone through emergency surgeries while Belay Bizuneh et al. [35] reported low levels of satisfaction influenced by other factors such as American Society of Anesthesiologists (ASA) status and the presence of pain immediately after operation.

Xu et al. [36] and Stambough et al. [37] explored the implications of opioid use on patient satisfaction. Xu et al. noted that opioid prescribing at surgical discharge is not associated with reduced patient satisfaction but with increased risk of presentation to healthcare due to side effects [36]. Stambough et al. added that more postoperative narcotic refills after total joint arthroplasty do not improve patient satisfaction and preoperative opioid and benzodiazepine use are associated with prolonged narcotic use [37]. Benavent et al. reported that patients consumed fewer opioid pills than prescribed and those who took more opioid pills had higher pain scores and lower satisfaction [38]. Similarly, Alema et al. highlighted the need for future in-depth studies on patient satisfaction and the lack of inclusiveness of non-pharmaceutical interventions in pain management strategies [39]. Fetene et al. found that overall patient satisfaction toward perioperative anesthesia service was low, indicating room for improvement in pain management practices [20].

Discussion

The integrative review shows the critical interaction of postoperative pain management, sleep quality, and patient satisfaction. Effective postoperative pain management is essential not just for pain relief but also for sound sleep quality, which is critical for better recovery and well-being of patients.

Postoperative Pain and Sleep Quality

A number of studies have shown that poorly managed postoperative pain disrupts sleep quality to a great extent. For example, Mouch et al. [21] and Kulpatcharapong et al. [12] revealed that postoperatively, pain and other modifiable factors such as light exposure may contribute to poor sleep quality. These observations are in line with more general literature that posits that pain can disrupt sleep and can bring about fragmented and less restorative sleep. Earlier studies by Chou et al. demonstrated that postoperative pain can cause poor quality of sleep, which in turn can encourage sensitivity to pain and thus create a circular pattern of poor sleep and hyperalgesia [40].

Pharmacological treatments have shown positive results in both conditions. Studies by Shakya et al. [22] and Fu et al. [23] have already proved that zolpidem or tramadol can improve sleep quality and concomitantly reduce pain. Such a dual benefit will be significant because restorative sleep might accelerate the process of healing while at the same time reducing the overall perception of pain as suggested by Tang et al. [41]. Enhancing preoperative sleep has demonstrated effectiveness in reducing postoperative pain as a programmed intermittent epidural bolus injection strategy during surgery works to reduce postoperative pain. These two strategies underline an all-encompassing approach to pain management from the preoperative, intraoperative, and postoperative phases. Non-pharmacologic approaches including cognitive behavioral therapy for insomnia (CBT-I) have been proven effective in managing sleep disturbances among patients suffering from chronic pain and therefore may offer an edge in the postoperative period among the same patients [41].

Satisfaction with Pain Management

Patient satisfaction is an outcome dependent on the methods of control management of side effects and personalization of strategies used for pain. According to Berkowitz et al., the link between clinical outcomes and patient satisfaction stands strong meaning effective pain management remains a crucial determinant of patient satisfaction [30]. This finding supports the study by Rahmqvist and Bara, who found that pain management quality is directly proportional to overall patient satisfaction within hospital settings [42]. Effective action regarding the personalization of pain management strategies has been reflected as a point that increases levels of patient satisfaction in several studies. For example, Peng et al. showed that a stratified approach to pain management developed from risk assessment levels increased analgesic success levels and postoperative recovery increasing patient satisfaction [31]. Effective pain management was successful using a multimodal method in patients after arthroscopic rotator cuff repair as seen in the study by Garvey et al. [32]. These findings agree with those of Chou et al., who also put forward multimodal analgesia as the optimal choice for adequate pain control because it reduces the intensity of pain and the use of opioids [40].

Nevertheless, as shown in various studies, patient satisfaction with pain management is still rated at a low level in several areas. Yunus et al. [34] and Liu et al. [29] found significant inadequacies in the satisfaction of patients from pain management. Both studies showed the necessity for developing universal protocols for the management of pain which could be implemented in various healthcare settings. According to Gan et al. [43], despite achievements that have been made in pain management after surgery there are too many cases in which patients feel inadequate anesthesia. Of particular significance is that opioid use impacts the satisfaction of the patient. Most of such patients are discharged with an opioid prescription that increases the risk of side effects which reduces patient satisfaction. This calls for balanced strategies in pain management with minimal use of opioids in favor of multimodal approaches in the management of pain. Hill et al. support this showing that reducing opioid prescriptions does not compromise pain control and can improve overall patient satisfaction [44].

## Conclusions

The current review emphasizes the need to create and implement effective and all-inclusive pain management protocols that will decrease the intensity of pain, improve sleep quality, and assure the satisfaction of the patient. The implementation of such protocols is likely to result in better postoperative outcomes, reduced hospital stays, and overall improved patient experience. Healthcare providers can significantly improve their quality of care for postoperative patients when the interconnection of pain, sleep, and satisfaction is addressed.

Future research should build and implement comprehensive protocols for managing pain that look at the multi-facets of pain and its effects on sleep quality and satisfaction. In this way, it is necessary to have protocols that combine the pharmacological and non-pharmacological interventions according to the needs of each patient. More studies should be done to look at how improved sleep might have long-term consequences relative to the postoperative recovery and satisfaction of patients. The level of postoperative care offered across regions will be standardized if the application of the standard guidelines in managing pain becomes a reality in all areas through the training of health workers involved in care delivery and monitoring. With such training, there will be improvements in the general quality of care.
